# Diversity and Phylogenetic Analyses of Bacterial Symbionts in Three Whitefly Species from Southeast Europe

**DOI:** 10.3390/insects8040113

**Published:** 2017-10-20

**Authors:** Marisa Skaljac, Surapathrudu Kanakala, Katja Zanic, Jasna Puizina, Ivana Lepen Pleic, Murad Ghanim

**Affiliations:** 1 Department of Applied Sciences, Institute for Adriatic Crops, Put Duilova 11, Split 21000, Croatia; katja.zanic@krs.hr; 2 Department of Entomology, Agricultural Research Organization, Institute of Plant Protection, the Volcani Center, Bet Dagan 50250, Israel; kanakalavit@gmail.com (S.K.); ghanim@volcani.agri.gov.il (M.G.); 3 Fraunhofer Institute for Molecular Biology and Applied Ecology (IME), Bioresources Project Group, Winchesterstrasse 2, 35394 Giessen, Germany; 4 Faculty of Science, University of Split, Rudera Boskovica 33, Split 21000, Croatia; jasna.puizina@pmfst.hr; 5 Laboratory for Aquaculture, Institute of Oceanography and Fisheries, Setaliste Ivana Mestrovica 62, Split 21000, Croatia; lepen@izor.hr

**Keywords:** bacterial symbionts, phylogenetic analyses, mtCOI, diversity, whiteflies

## Abstract

*Bemisia tabaci* (Gennadius), *Trialeurodes vaporariorum* (Westwood), and *Siphoninus phillyreae* (Haliday) are whitefly species that harm agricultural crops in many regions of the world. These insects live in close association with bacterial symbionts that affect host fitness and adaptation to the environment. In the current study, we surveyed the infection of whitefly populations in Southeast Europe by various bacterial symbionts and performed phylogenetic analyses on the different symbionts detected. *Arsenophonus* and *Hamiltonella* were the most prevalent symbionts in all three whitefly species. *Rickettsia* was found to infect mainly *B. tabaci*, while *Wolbachia* mainly infected both *B. tabaci* and *S. phillyreae.* Furthermore, *Cardinium* was rarely found in the investigated whitefly populations, while *Fritschea* was never found in any of the whitefly species tested. Phylogenetic analyses revealed a diversity of several symbionts (e.g., *Hamiltonella*, *Arsenophonus*, *Rickettsia*), which appeared in several clades. Reproductively isolated *B. tabaci* and *T. vaporariorum* shared the same (or highly similar) *Hamiltonella* and *Arsenophonus*, while these symbionts were distinctive in *S. phillyreae*. Interestingly, *Arsenophonus* from *S. phillyreae* did not cluster with any of the reported sequences, which could indicate the presence of *Arsenophonus*, not previously associated with whiteflies. In this study, symbionts (*Wolbachia*, *Rickettsia*, and *Cardinium*) known to infect a wide range of insects each clustered in the same clades independently of the whitefly species. These results indicate horizontal transmission of bacterial symbionts between reproductively isolated whitefly species, a mechanism that can establish new infections that did not previously exist in whiteflies.

## 1. Introduction

Insects share a long-term and intimate association with bacterial symbionts, which has helped their hosts to become well adapted to numerous challenges present in their environment [1,2,3,4]. Primary bacterial symbionts are essential for insects to live on nutritionally poor diets, while secondary bacterial symbionts can additionally affect traits such as development, reproduction, thermal tolerance, protection from natural enemies, and immunity [2,5,6,7,8,9].

Whitefly species such as *Bemisia tabaci* (Gennadius), *Trialeurodes vaporariorum* (Westwood), and *Siphoninus phillyreae* (Haliday) are phloem-feeding pests that damage numerous agricultural crops [10,11,12,13]. In particular, *B. tabaci* and *T. vaporariorum* are globally distributed, extremely polyphagous, and cause indirect damage by transmitting important plant viruses [14,15,16]. All three whitefly species are invasive and have a long history of resistance to insecticides from different chemical classes [13,17,18]. *B. tabaci* presents a species complex, composed of at least 40 morphologically indistinguishable species [10,15,19,20,21,22,23,24,25,26,27]. *B. tabaci species* Middle East-Asia Minor 1 (MEAM1) and Mediterranean (MED) are globally distributed and the most challenging to control [15,28,29,30]. The high diversity in mitochondrial cytochrome oxidase I (mtCOI) gene sequences revealed differentiation within *B. tabaci* (MED) into four groups: Q1 (western Mediterranean populations), Q2 (Middle Eastern populations), Q3 (Burkina Faso populations), and Africa Silver Leafing (ASL) (Ivory Coast, Burkina Faso, and Cameroon) [31,32,33].

All whitefly species harbor a primary symbiont, *Portiera aleyrodidarum*, which supplements the insects’ diet with essential amino acids and carotenoids [34]. In *B. tabaci*, several bacterial symbionts have been described, including *Hamiltonella*, *Rickettsia*, *Wolbachia*, *Cardinium*, *Arsenophonus*, and *Fritschea* [35,36,37,38]. Several studies have attempted to link infection with the secondary symbiotic community with *B. tabaci* speciation and performance [33,38]. However, due to horizontal transmission, this is difficult to prove [33,39,40].

Diverse secondary symbionts have been found in *T. vaporariorum* and *S. phillyreae* populations as well [39,41]. *T. vaporariorum* was infected with *Hamiltonella*, *Rickettsia*, *Wolbachia*, *Cardinium*, and *Arsenophonus*, while *S. phillyreae* contained the same symbiotic composition as *T. vaporariorum* with no detected presence of *Rickettsia*. *Wolbachia*, *Rickettsia*, *Cardinium*, and *Arsenophonus* are known to manipulate host reproduction in many insect species [2,42,43,44]. In *B*. *tabaci*, *Rickettsia* induces the expression of genes required for thermotolerance [45], provides the host with fitness benefits [46], increases its susceptibility to insecticides [47], and is involved in hosts’ response to parasitism [48]. *Arsenophonus* was additionally found to act as an obligatory mutualist in lice [49,50]. The GroEL proteins produced by *Arsenophonus* in *B*. *tabaci* (Asia II species) and *Hamiltonella* in Israeli populations of *B. tabaci* (MEAM1) were found to interact with the coat protein of begomovirus and therefore facilitate virus transmission [51,52]. The GroEL produced by other symbionts of *B. tabaci* (MEAM1 and MED) did not interact with the virus and therefore were not involved in virus transmission [52]. Furthermore, in the pea aphid, *Hamiltonella* confers resistance to parasitoids and increases thermotolerance [53,54]. Recently, the genome of *Hamiltonella* from *B. tabaci* (MED) was sequenced, and this dataset provided insights into nutritional benefits this symbiont may provide to the whitefly host [55,56]. *Fritschea* has been reported only in the New World *B. tabaci* species, and at present, nothing is known concerning its function [57,58].

The focus of this and our previous studies were whiteflies from Southeast Europe (Croatia, Montenegro, Bosnia and Herzegovina, Macedonia, and Serbia) and their associated bacterial symbionts. In this region, *B. tabaci*, *T. vaporariorum*, and *S. phillyreae* have been identified and possibly predominate [39,41]. *T. vaporariorum* has been reported as the predominant whitefly species in the surveyed region, while *B. tabaci* and *S. phillyreae* have never been reported from Serbia [41] (J. Bocanski, pers. comm.). Based on molecular markers, two invasive species of *B. tabaci* were detected; in Montenegro, both the MEAM1 and MED species were detected, whereas in Croatia and Bosnia and Herzegovina, only the MED species was confirmed [39,41]. Q1 and Q2 groups of *B. tabaci* (MED) were reported from Croatia and Montenegro [19,41]. All three whitefly species had unique co-infections with bacterial symbionts compared to previously reported studies [28,37,58]. Our aim was to add further details concerning the global phylogenetic diversity of bacterial symbionts between the three whitefly species. This may give further insights about the functions these symbionts may have in whitefly biology.

## 2. Materials and Methods

### 2.1. Whitefly Populations

**Figure 1:**
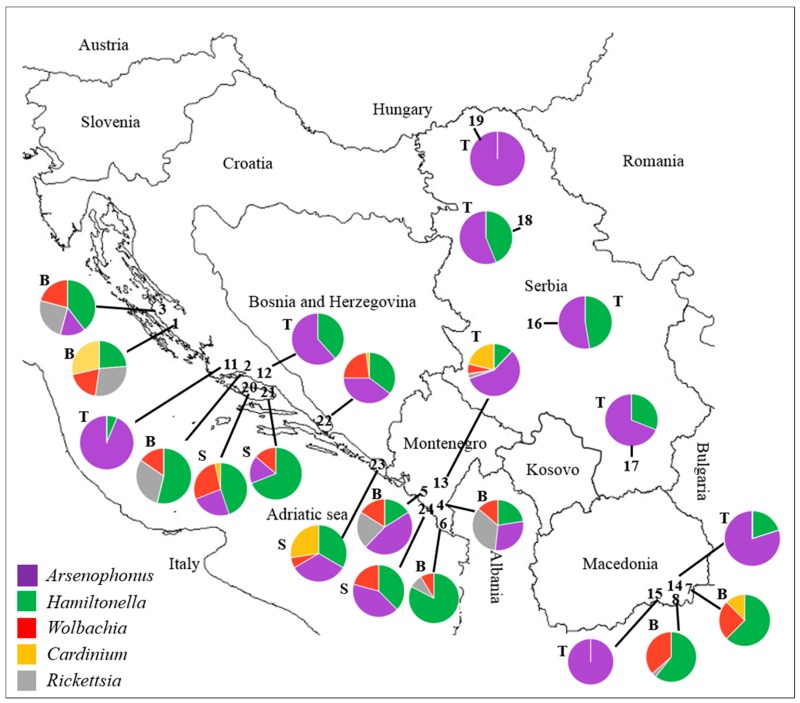
Locations of collected whitefly populations (according to population numbers in Table S1) with overview of overall infection frequencies of secondary bacterial symbionts (according to Table 2) in the three whitefly species in Southeast Europe. Each chart is marked with a code (B, T, and S) in order to distinguish between the whitefly species. B, *Bemisia tabaci*; T, *Trialeurodes vaporariorum*; S, *Siphoninus phillyreae*.

Populations of the three whitefly species were collected in the summers from 2009 to 2013 in regions with intense agricultural activity in Croatia, Montenegro, Macedonia, and Serbia (Figure 1, Table S1). Whitefly specimens were collected from open fields or greenhouses when high population densities were available. Sampling locations, whitefly species, and host plants are shown in Table S1 and Table 3. Whitefly populations from Macedonia and Serbia were newly examined; two laboratory populations of *B. tabaci* from Israel were used for comparison, whereas other whitefly populations from Croatia and Montenegro were previously screened in Skaljac et al. (2010, 2013) [39,41] and used in this study as well. Adults of the three whitefly species were collected using a Pasteur pipette attached to a hand-held aspirator and then moved into absolute ethanol and kept at −20 °C until processing.

### 2.2. Screening and Sequencing of Secondary Bacterial Symbionts and Molecular Identification of B. tabaci Species

Adults (*n* = 10–20 per population) from the whitefly populations (Table S1) were tested for the presence of secondary symbionts and *B. tabaci* species (MED or MEAM1) identification. Genomic DNA of each individual was extracted in lysis buffer as previously described in Chiel et al. (2007) [37] and used for further screening. *Bemisia tabaci* MED or MEAM1 groups were identified using microsatellite markers by polymerase chain reaction (PCR) amplification using Bem 23 primers and fragment sizes. The product size obtained from MEAM1 was 200 bp, and from the MED, it was 400 bp. In addition, we amplified mtCOI gene fragment for phylogenetic analyses of *B. tabaci* species (Table 1). Secondary bacterial symbionts were tested by PCR using genus-specific primers amplifying 16*S* or 23*S* rDNA (Table 1). We did not detect *Fritschea* in our previous studies, and due to the lack of a positive control, *Fritschea* was not considered in this study.

PCR was carried out in 25 μL volumes containing 4 μL DNA lysate, 10 pmol of each primer, 10 mM dNTP mix, 10× PCR Rxn Buffer, 2 mM MgCl_2_, and 5 units/μL of Platinum Taq DNA polymerase (Invitrogen). PCR products were visualized on a 1.5% agarose gel containing ethidium bromide. The PCR products were eluted, and DNA was ligated into a pGEM-T Easy Vector (Promega, Madison, WI, USA) following the manufacturer’s instructions. After transfection into RapidTrans TAM1 competent *E. coli* (Active Motif, Carlsbad, CA, USA), cells were grown on LB agar (Invitrogen) at 37 °C overnight. Clones were then screened for PCR inserts using the primers listed in Table 1. Colonies were then grown overnight in 5 mL LB medium containing ampicillin (100 µg/mL) in a shaking incubator at 200 rpm and 37 °C. Plasmid DNA from independent colonies was purified using a QIAprep Spin Miniprep kit (Qiagen, Hilden, Germany) and sent for sequencing (3730xl DNA analyzer, Macrogen Europe, Amsterdam, The Netherlands). The sequences were obtained from 10–20 individual specimens per primer set and compared with those in the databases using the BLAST algorithm at the NCBI [63].

### 2.3. Phylogenetic Analyses

Mitochondrial COI and secondary symbiont sequences for all *B. tabaci*, *T. vaporarium*, and *S. phillyreae* obtained during this study, together with reference sequences from GenBank, were aligned using the program MAFFT 7 [64] followed by visual inspection and manual adjustment. The appropriate model of evolution was estimated using the Bayesian Information Criterion (BIC) with Jmodeltest 2.1.6 on CIPRES Science Gateway [65], and selected models were the same for ML inferences. Analyses were initiated from random starting trees. The models selected were GTR+G for mtCOI, HKY for *Hamiltonella*, GTR+G for *Arsenophonus* and *Wolbachia*, and HKY+I for *Cardinium* and *Rickettsia*. Using these models, phylogenetic reconstruction was carried out using Bayesian inference (BI). For BI analysis, two independent runs of Markov Chain Monte Carlo (MCMC) were done for 10 million generations using eight chains and a sampling frequency of 1000 generations by MrBayes ver. 3.2 [66]. The first 25% of the samples were discarded by setting the burn-in fraction to 0.25. This burn-in setting was shared across the SUMT command to discard 25% of sampled trees. These phylogenetic trees were viewed by FigTree V. 1.4.2, available at http://tree.bio.ed.ac.uk/software/figtree/. Pairwise genetic distances for whitefly mtCOI sequences were analyzed using the p-distance model of evolution in MEGA 6.0 [67]. The whitefly sequences characterized in this study were assigned to different species on the basis of the rule of >3.5% pairwise sequence divergence [19].

### 2.4. Nucleotide Sequence Accession Numbers

The nucleotide sequences of the genes identified in this study have been deposited in GenBank under the accession numbers KY623456 to KY623475 for *Arsenophonus*, from KY621483 to KY621519 for *Hamiltonella*, from KY994554 to KY994569 for *Rickettsia*, from KY994545 to KY994553 for *Wolbachia*, from KY994541 to KY994544 for *Cardinium*, and from KY628431 to KY628448 for mtCOI from *B. tabaci*.

## 3. Results

### 3.1. Whitefly Infection with Secondary Bacterial Symbionts

Ten to 20 individuals from each population of the three whitefly species were tested for the presence of the secondary bacterial symbionts. Infection frequencies with multiple secondary symbionts present in 24 whitefly populations used in this and our previous studies are given in Table 2 and Table 3, and Figure S1. All the tested individuals were positive for the primary symbiont of whiteflies, *P. aleyrodidarum*, and this was considered to control the quality of extracted DNA.

We could not find a correlation between infection with bacterial symbionts and host plants that whiteflies infested, as was suggested in our previous studies (Table 2) [39,41].

#### 3.1.1. *Bemisia tabaci* Infection with Secondary Bacterial Symbionts

*B. tabaci* populations were collected in Croatia, Montenegro, and Macedonia (Figure 1; Table S1). All populations used in this study were identified as MED species and they belonged to groups Q1 and Q2 (Table 2 and Table 3). These populations had high prevalence of mixed infections with *Hamiltonella*, *Arsenophonus*, *Rickettsia*, *Wolbachia*, and *Cardinium* (Table 2). *Hamiltonella* was highly prevalent or fixed in *B. tabaci* MED (Q1) populations from Macedonia (Doiran, Gevgelija) and Montenegro (Darza). *Arsenophonus* was prevalent in populations from Montenegro (Bar—MED Q2, Lastva Grbaljska—MED Q1), whereas *Rickettsia* was fixed in population collected in Bar (MED Q2). Individuals of both Q1 and Q2 groups of *B. tabaci* MED were mainly infected with one (5–75%) or two symbionts (15–35%), whereas infections with three and four symbionts were only common in Q2 *B. tabaci* MED populations from Zadar (Croatia) and Bar (Montenegro) (Table 3). The two populations of *B. tabaci* MED from Croatia (Turanj-Q2, Split-Q1) had 35% of individuals that did not contain any of the tested secondary symbionts. *Rickettsia* infected around 70–75% of individuals of both Israeli *B. tabaci* MED (Q2) and MEAM1 populations. Additionally, *Arsenophonus* was fixed in Israeli MED (Q2), whereas *Hamiltonella* was fixed in MEAM1 population. Beside *Rickettsia* and *Arsenophonus*, around 20% of individuals of the Israeli MED (Q2) population were infected with *Wolbachia*. All individuals of both Israeli MED (Q2) and MEAM1 populations contained at least one secondary symbiont. In Israeli MED (Q2), 25% of individuals were infected with one, 55% with two symbionts, and 20% of individuals with three symbionts. Israeli MEAM1 population contained individuals infected with one (30%) and two (70%) secondary symbionts.

#### 3.1.2. *Trialeurodes vaporariorum* Infection with Secondary Bacterial Symbionts

*T. vaporariorum* populations were collected in Croatia, Montenegro, Macedonia, and Serbia (Figure 1 and Table S1). All populations used in this study were predominantly infected with *Hamiltonella* and *Arsenophonus* (Table 2). The only exception was *T. vaporariorum* population collected in Montenegro (Podgorica), which was additionally infected with *Rickettsia*, *Wolbachia*, and *Cardinium*. *Arsenophonus* was fixed or close to fixation in the populations collected in Montenegro, Macedonia, and Serbia, while the infection rate for *Hamiltonella* varied between 5% and 90% independently of the location (Table 2). Individuals of *T. vaporariorum* were mainly infected with one (10–100%) or two symbionts (5–90%), whereas there were no infections detected with three or four symbionts. The populations of *T. vaporariorum* from Croatia (Split) and Serbia (Opovo, Zorka Subotica) had 5–25% of individuals that did not contain any of the tested secondary symbionts.

#### 3.1.3. *Siphoninus phillyreae* Infection with Secondary Bacterial Symbionts

*S. phillyreae* populations were collected in Croatia and Montenegro (Figure 1 and Table S1). All populations used in this study had high prevalence of mixed infections with *Hamiltonella*, *Arsenophonus*, *Wolbachia*, and *Cardinium* (Table 2). Interestingly, *Rickettsia* was not found in any of the tested populations. Both *Arsenophonus* and *Hamiltonella* were highly prevalent in all the tested *S. phillyreae* populations (25–100% and 65–100%, respectively). All the tested *S. phillyreae* populations were infected with *Wolbachia* (15–55%), while *Cardinium* was detected only in populations from Croatia (Supetar, Pucisca, Ljuta). Individuals of *S. phillyreae* were commonly infected with one (5–60%), two (20–50%), and three symbionts (5–55%) (Table 3). Infections with four symbionts were rare and appeared only in individuals from Croatian *S. phillyreae* populations from Opuzen and Ljuta in 5% and 10%, respectively. Overall, all *S. phillyreae* individuals contained at least one secondary symbiont.

### 3.2. Phylogenetic Relationships of *B. tabaci* Sequences

**Figure 2:**
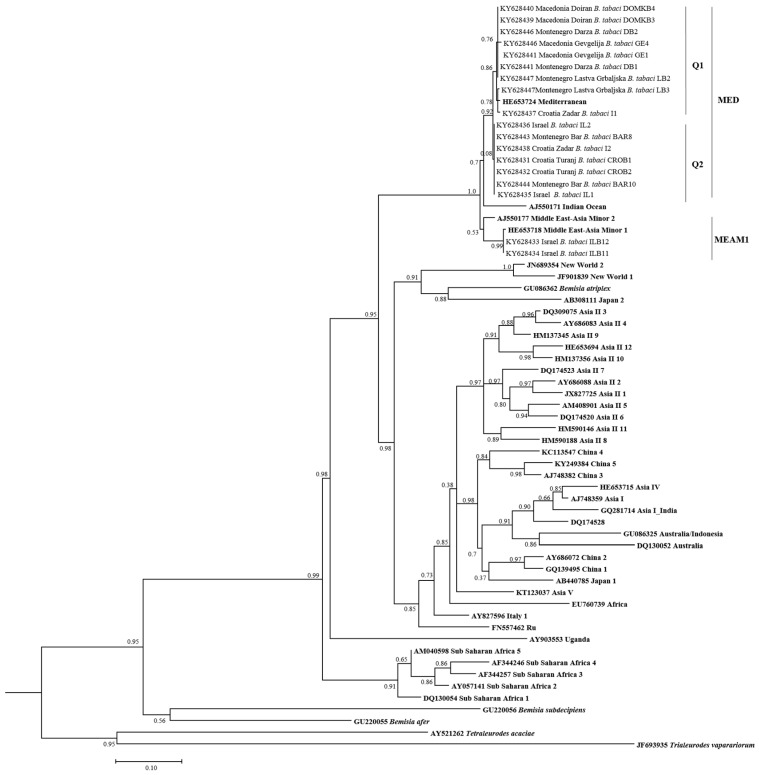
Phylogenetic tree based on the Bayesian analysis of 657 bp of mtCOI gene sequences of *Bemisia tabaci* populations. Sequences from GenBank are indicated in bold, while those obtained in this study are rearranged based on the geographical origin of *B. tabaci* individuals. Posterior probabilities are indicated at nodes.

A total of 18 *B. tabaci* sequences were characterized from Croatia, Montenegro, and Macedonia. In this study, *B. tabaci* sequences from Israeli populations served as a reference. A phylogenetic tree was constructed from the mtCOI sequences of *B. tabaci* and divergence analyses were compared with 40 putative species previously reported [10,15,19,20,21,22,23,24,25,26,27] (Figure 2). Our results showed that *B. tabaci* populations from Croatia, Montenegro, Macedonia, and Israel were identified as MED (Q1 and Q2) species. Populations of *B. tabaci* MED from Macedonia (Doiran, Gevgelija), Montenegro (Darza, Lastva Grbaljska), and Croatia (Split) belonged to the Q1 group, whereas populations from Croatia (Turanj) and Montenegro (Bar) belonged to the Q2 group (Figure 2). The exception was a *B. tabaci* MED population from Croatia (Zadar), which contained individuals from both Q1 and Q2 groups (Figure 2). In addition, the *B. tabaci* MEAM1 species was identified from the Israeli population only.

### 3.3. Phylogenetic Analysis of Secondary Symbionts in Whiteflies

**Figure 3:**
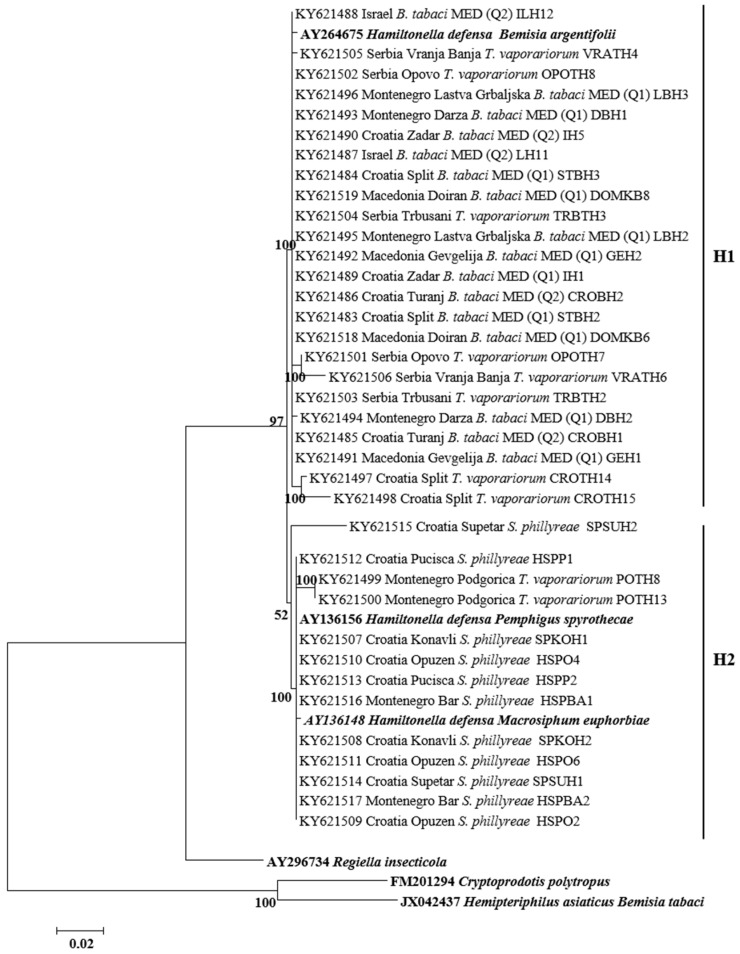
Molecular phylogenetic placements of *Hamiltonella* from whitefly species. The tree was constructed via Bayesian inference (BI) using an HKY substitution model. Sequences from GenBank are indicated in bold, while those obtained in this study are rearranged based on geographical origin and associated whitefly hosts, *B. tabaci*, *T. vaporariorum*, and *S. phillyreae*.

**Figure 4:**
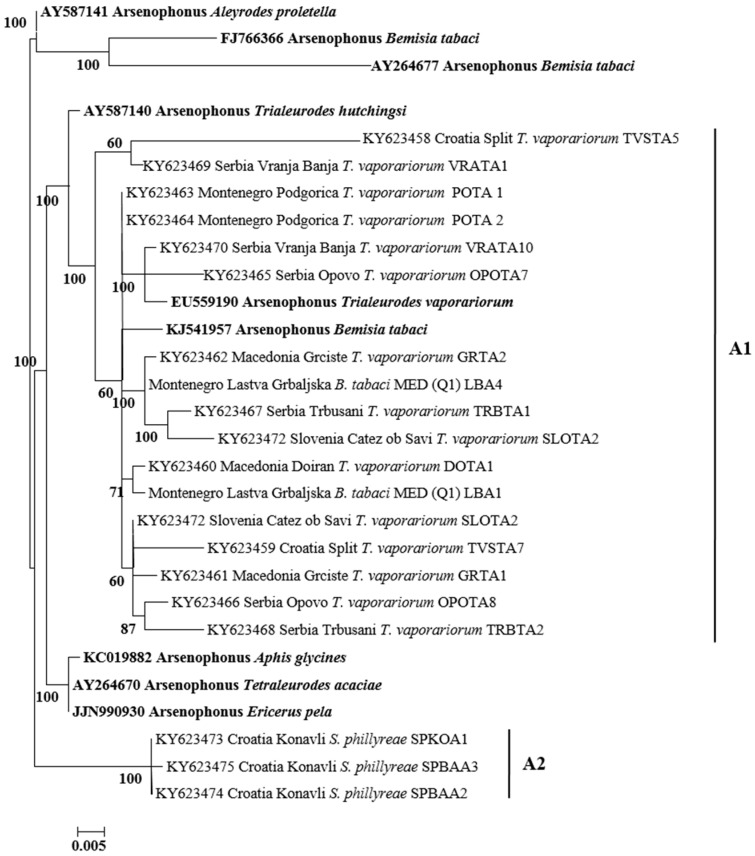
Molecular phylogenetic placements of *Arsenophonus* from whitefly species. The tree was constructed via Bayesian inference (BI) using a TPM1 µf substitution model. Sequences from GenBank are indicated in bold, while those obtained in this study are rearranged based on geographical origin and associated whitefly hosts, *B. tabaci*, *T. vaporariorum*, and *S. phillyreae*.

**Figure 5:**
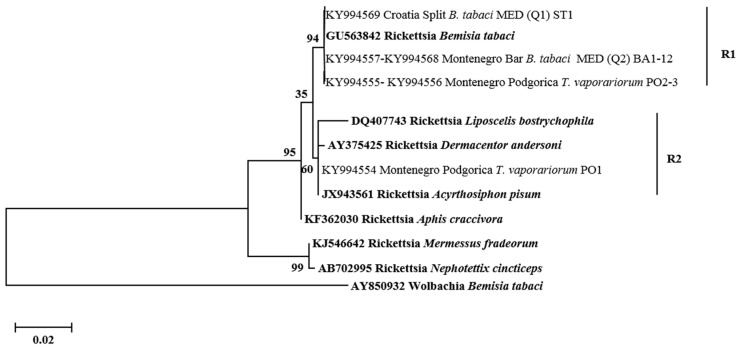
Molecular phylogenetic placements of *Rickettsia* from whitefly species. The tree was constructed via Bayesian inference (BI) using a GTR+G substitution model. Sequences from GenBank are indicated in bold, while those obtained in this study are rearranged based on geographical origin and associated whitefly hosts, *B. tabaci* and *T. vaporariorum*.

**Figure 6:**
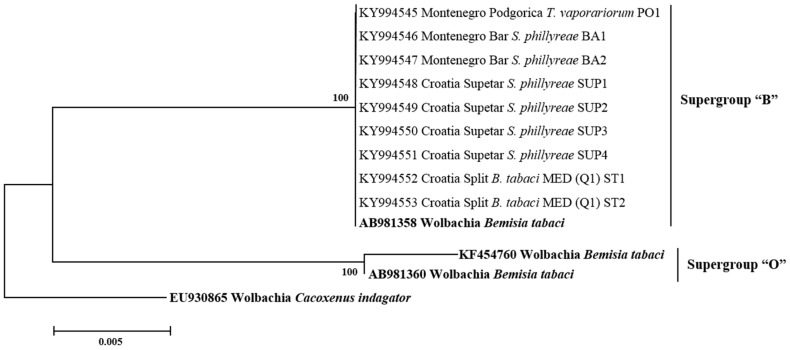
Molecular phylogenetic placements of *Wolbachia* from whitefly species. The tree was constructed via Bayesian inference (BI) using a GTR+G substitution model. Sequences from GenBank are indicated in bold, while those obtained in this study are rearranged based on geographical origin and associated whitefly hosts, *B. tabaci*, *T. vaporariorum*, and *S. phillyreae*.

**Figure 7:**
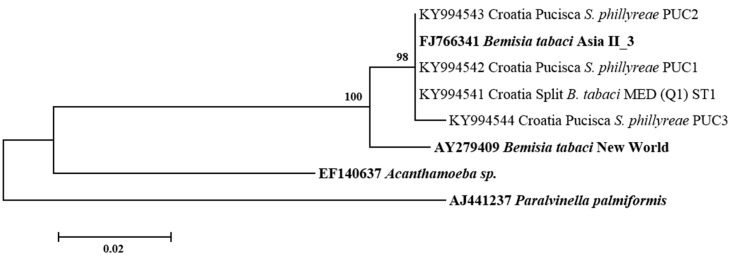
Molecular phylogenetic placements of *Cardinium* from whitefly species. The tree was constructed via Bayesian inference (BI) using an HKY+I substitution model. Sequences from GenBank are indicated in bold, while those obtained in this study are rearranged based on geographical origin and associated whitefly hosts, *B. tabaci* and *S. phillyreae*.

All secondary symbionts sequenced in this study were compared with bacterial reference sequences from GenBank (https://www.ncbi.nlm.nih.gov/genbank/). Sequence analysis revealed that, in addition to species diversity, several bacterial subgroups/strains may exist within the different bacterial genera tested. For *Hamiltonella*, the sequences from *B. tabaci*, *T. vaporarium*, and *S. phillyreae* formed two groups (H1 and H2) (Figure 3). Clade H1 contains *Hamiltonella* from *B. tabaci* and *T. vaporarium* from Croatia, Montenegro, Serbia, and Macedonia. Group H2 contains *Hamiltonella* from *S. phillyreae* and *T. vaporarium* from Croatia and Montenegro, respectively. 23rDNA sequences of *Arsenophonus* cluster into two major groups A1 and A2 (Figure 4). The A1 group contains strains from *B. tabaci* and *T. vaporarium*. Group A2 contains a distinct *Arsenophonus* strain from *S. phillyreae* from Croatia and *T. vaporarium* from Montenegro, which differs from the A1 group. *Rickettsia* was grouped into two major clusters, R1 and R2 (Figure 5). The top clade (R1) consists of *Rickettsia* from both *B. tabaci* and *T. vaporarium* from Croatia and Montenegro. Clade R2 consists of *Rickettsia* from only *T. vaporarium* from Montenegro. Interestingly, none of the *S. phillyreae* individuals tested positive for *Rickettsia.* In the case of *Wolbachia*, to date, 16 supergroups successively named A–Q infecting a wide range of arthropods and filarial nematodes have been reported [68]. In our analysis, the B supergroup was observed in all three species tested from Croatia, Montenegro, and Macedonia (Figure 6). 16*S* rDNA sequences of *Cardinium* from *B. tabaci* and *S. phillyreae* are grouped under a single clade (Figure 7).

## 4. Discussion

Numerous studies have shown that whitefly-associated secondary bacterial symbionts (*Arsenophonus*, *Hamiltonella*, *Rickettsia*, *Wolbachia*, and *Cardinium*) play major roles in whitefly biology and have developed through evolutionary history numerous mechanisms to invade and persist in the insect host [1,45,46,52,69,70]. Besides common secondary symbionts, several studies reported the presence of other bacteria in *B. tabaci* (e.g., *Bacillus*, *Enterobacter*, *Paracoccus*, and *Acinetobacter*) [71,72]. However, so far none of those bacteria have established a relationship with whiteflies as secondary symbionts investigated in this study. All secondary symbionts are vertically transmitted with high fidelity, but most of them are frequently transferred via horizontal transmission routes, allowing them to spread successfully within and between the species [73].

Our recent studies have extensively investigated infection and localization of secondary symbionts in the three whitefly species (*B. tabaci*, *T. vaporariorum, S. phillyreae*) in Southeast Europe [39,41]. We found unique co-infection patterns, revealing the presence of similar secondary symbionts in reproductively isolated whiteflies. This occurrence is likely to be due to highly dynamic processes of horizontal transfer of symbionts between insect hosts, possibly via sharing host plants, mating, or parasitoids [38,73,74]. These events are necessary for the symbiont to spread to novel hosts [75]. Such acquisition of bacterial symbionts can directly impact host biology [46]. For example, the spread of *Rickettsia* in *B. tabaci* from the United States resulted in large fitness benefits and female bias in the tested whiteflies [46].

Intensive plant trade occurs between Mediterranean countries and this provides opportunities for introducing new whitefly species and symbionts in regions where they have not existed before [76]. Several studies reported the invasion of *B. tabaci* MED (Q2), previously associated only with the Middle East countries of the Mediterranean basin, into Spain, France, and Italy, where it coexisted with the native *B. tabaci* MED (Q1) [76,77]. Our study revealed the presence of both Q1 and Q2 groups of *B. tabaci* MED in the investigated region (Table 2, Figure 2). Two populations identified as Q2 were collected in Croatia (Turanj) and Montenegro (Bar). Interestingly, a *B. tabaci* MED population (Croatia-Zadar) collected on plants imported from Italy consisted of both Q1 and Q2 groups and they carried *Arsenophonus*, which was not previously detected in existing *B. tabaci* MED (Q1) populations (Table 2, Figure 2) [39].

Variations in the diversity and infection frequencies of bacterial symbionts between the three whitefly species in this study were substantial (Table 2 and Table 3, Figure S1). 

Our results revealed that *Arsenophonus* and *Hamiltonella* from both *B. tabaci* and *T. vaporariorum* were phylogenetically grouped in the same clades (with several exceptions; e.g., *Hamiltonella* from *T. vaporariorum* Podgorica), compared with those symbionts from *S. phillyreae*, which were placed in more distant clades (Figure 3 and Figure 4). *B. tabaci* and *T. vaporariorum* frequently share host plants, therefore the possibility of horizontal transmission of bacterial symbionts between these two whitefly species is high [39]. *S. phillyreae* was only found on pomegranate plants, which was not a host for the other two whitefly species [40,42]. Placement of *Hamiltonella* in different clades was also reflected on its localization pattern in the whitefly hosts. *Hamiltonella* found in *B. tabaci* and *T. vaporariorum* was localized exclusively in bacteriosomes, while in *S. phillyreae* it was found to be scattered throughout the entire bodies of nymphs and adults [39,41].

*Hamiltonella* was present at high frequencies in the investigated species of *B. tabaci* MED (Q1) and MEAM1 (Table 2), suggesting it had a major impact on the host biology. The infection frequencies of *Hamiltonella* were in accordance with the global screening reports of bacterial symbionts in *B. tabaci* MED (Q1) and MEAM1 [33,38,40,78,79]. However, these studies showed absence of *Hamiltonella* in *B. tabaci* species such as MED (Q2, ASL), Sub-Saharan Africa 1–5, Asia II, and China 1 [33,78,80]. This was contrary to our results, where *Hamiltonella* infected 25% and 65% of individuals from MED (Q2) populations from Croatia (Turanj) and Montenegro (Bar), respectively (Table 2). Infection of *Hamiltonella* varied in *T. vaporariorum* populations (from absence of infection to high prevalence) in this study, whereas infection with this symbiont was highly prevalent in *S. phillyreae*. Surprisingly, a large study by Kapantaidaki et al. (2014) [81] did not report infection of *Hamiltonella* in *T. vaporariorum* populations from different global locations.

In general, the infection frequencies of *Arsenophonus* in *B. tabaci* varied greatly and sometimes depended on the genetic group. *B. tabaci* MEAM1 had low infection rates with *Arsenophonus* [33,38,39,40]. This was also similar to several *B. tabaci* (MED) populations from Southeast Europe (Q1) and China, but some MED genetic groups from Montenegro (Q1), Israel (Q2), and Burkina Faso (Q3) were highly infected with *Arsenophonus* [33,39,41,78]. This variable occurrence of *Arsenophonus* in *B. tabaci* is in agreement with our results. For example, *Arsenophonus* was fixed in *B. tabaci* MED (Q2) from Israel and prevalent in MED (Q1 and Q2) from Montenegro (Lastva Grbaljska, Bar) (85% infection rate). However, this symbiont was absent in other MED (Q1 and Q2) populations (Table 2). *Arsenophonus* was constantly present at high frequencies in *T. vaporariorum* and *S. phillyreae* in our and other studies [39,40,41,81].

In this study, *Rickettsia* was present in nearly all populations of *B. tabaci* MED. However, infection rates were much lower independently of Q1 and Q2 groups compared to Israeli MED and MEAM1 populations (Table 2). This suggests recent introduction of *Rickettsia* in *B. tabaci* populations in the investigated region. Other studies have shown that *Rickettsia* was present at high infection rates in the MEAM1, MED (Q2), and China 1 groups of *B. tabaci*, while lower infection rates were observed in Asia II and the MED groups of *B. tabaci* from Burkina Faso (Q3) and China [33,38,40,78,79]. This symbiont was rarely detected in *T. vaporariorum*, and no infection was found in *S. phillyreae* populations (Table 2) [41,81].

*Wolbachia* is one of the most commonly present symbionts in insects [82]. In this study, nearly all *B. tabaci* and *S. phillyreae* populations were infected with *Wolbachia* (10–60% and 15–50%, respectively), while it was rarely found in *T. vaporariorum* (Table 2). Several studies reported highly diverse infection rates of *Wolbachia* in *B. tabaci* MED (Q1, Q2, ASL), while it was not observed in other genetic groups [33,40]. Bing et al. (2013) [78] found that native genetic groups of *B. tabaci* (Asia II, China 1) were highly infected with *Wolbachia*, whereas this symbiont was not detected in invasive MED and MEAM1 groups. *Cardinium*, another known reproductive manipulator, was rarely detected among populations of the three whitefly species in this study (Table 2) [39,41,43]. Other studies have revealed large differences in rates of *Cardinium* infection in populations from the *B. tabaci* species complex [33,38,40,78,79]. Phylogenetically, *Wolbachia* and *Cardinium* present in the three whitefly species from this study appeared in the same clades, together with other reported sequences of the same symbionts (Figure 6 and Figure 7).

Mixed infections and heterogeneity among populations in our study could be a result of competition for space and resources among the symbionts or the tolerance of the host to maintain diverse bacterial community [39,83]. This can contribute to multiple events of horizontal transmissions of secondary symbionts within the same whitefly species and reproductively isolated *B. tabaci* and *T. vaporariorum* (Table 2 and Table 3). In our study, horizontal transmission seems likely for *Hamiltonella*, *Arsenophonus*, *Wolbachia*, and *Cardinium* between *B. tabaci* and *T. vaporariorum*. *Rickettsia* was mostly associated with *B. tabaci*, and there were rare individuals from *T. vaporariorum* that were positive for this symbiont, which could reflect isolated events of horizontal transmission [41]. 

Chiel et al. (2009) [75] demonstrated horizontal transmission of *Rickettsia* in *B. tabaci* using parasitoid wasps, while transmission of *Hamiltonella* failed in this system. In addition, *Rickettsia* was successfully shown in *B. tabaci* to be transmitted via host plants [73]. There are evidences for horizontal transmission of bacterial symbionts such as *Arsenophonus*, *Cardinium*, and *Wolbachia* either among whiteflies, between whiteflies and arthropods present in the environment, or, as previously mentioned, via host plants [62,84,85,86]. Phylogenies of secondary symbionts are largely incongruent with those of their hosts, which is not the case with the phylogeny of primary symbionts [86]. This suggests an ancient infection of a host with primary symbiont followed by vertical transmission and consequent cospeciation with the insect host. In contrast, incongruent phylogenies suggest multiple infections and horizontal transfer between different hosts [86,87,88].

In the investigated bacterial community of the three whiteflies, we have found more than 20 different symbiotic combinations in a range from no infected individuals to those that were infected with four symbionts (Table 3). In populations of *B. tabaci* and *T. vaporariorum* single infections dominated (43% and 56%, respectively), while in *S. phillyreae* single (25%), double (38%), and triple (31%) infections were present in similar range. Double infections were present in 25% and 38% of individuals of *B. tabaci* and *T. vaporariorum*, respectively. Interestingly, *T. vaporariorum* in this study had no infections with three or four symbionts, while those infections existed in *B. tabaci* in 14% and 7.5%, respectively. In *B. tabaci*, *T. vaporariorum*, and *S. phillyreae*, individuals with no infections were present in 11%, 5%, and 3%, respectively. Our results were slightly different from study of Gueguen et al. (2010) [33], where infections of global populations of *B. tabaci* with two or more symbionts were common and double infections dominated (59%). Furthermore, several studies on sub-Saharan populations of *B. tabaci* report increasing number of secondary symbiont-free whiteflies [88,89]. It is likely to conclude that heterogeneity of symbiotic community of the whiteflies depends on location, population, and time [88].

## 5. Conclusions

The present study found substantial diversity of bacterial symbionts in the three whitefly species, especially between those that shared habitat (*B. tabaci* and *T. vaporariorum*) and in *S. phillyreae*, which was exclusively found on pomegranate plants. Dynamic processes of horizontal transmission could bring phylogenetically distant symbionts together in previously uninfected whiteflies (e.g., via parasitoids). This finding can contribute to the discovery of new functions that symbionts such as *Hamiltonella* or *Arsenophonus* may fulfill in their whitefly hosts.

## Figures and Tables

**Table 1:** The primers used in this study.

Primer Name	Sequence (5′→3′)	Annealing (°C)/Size (bp)	Gene	Reference
Bem 23 F Bem 23 R	CGGAGCTTGCGCCTTAGTC CGGCTTTATCATAGCTCTCGT	55/MEAM1 = 200; MED = 400	Microsatellite	[59]
C1-J-2195 L2-N-3014	TTGATTTTT TGGTCATCCAGAAGT TCCAATGCACTAATCTGCCATATTA	51/850	Cytochrome oxidase I (mtCOI)	[60]
Por-F Por-R	TGCAAGTCGAGCGGCATCAT AAAGTTCCCGCCTTATGCGT	59/1000	*Portiera* 16*S* rDNA	[35]
Rb F Rb R	GCTCAGAACGAACGCTATC GAAGGAAAGCATCTCTGC	59/900	*Rickettsia* 16*S* rDNA	[36]
92 F Hb R	TGAGTAAAGTCTGGGAATCTGG AGTTCAAGACCGCAACCTC	62/700	*Hamiltonella* 16*S* rDNA	[35]
Ars23S-1 Ars23S-2	CGTTTGATGAATTCATAGTCAAA GGTCCTCCAGTTAGTGTTACCCAAC	59/600	*Arsenophonus* 23*S* rDNA	[61]
Wol16S F Wol16S R	CGG GGGAAAAATTTATTGCT AGCTGTAATACAGAAAGTAAA	55/650	*Wolbachia* 16*S* rDNA	[38]
CFB F CFB R	GCGGTGTAAAATGAGCGTG ACCTMTTCTTAACTCAAGCCT	59/500	*Cardinium* 16*S* rDNA	[62]

**Table 2:** Overview of overall infection frequencies of secondary bacterial symbionts in the three whitefly species in Southeast Europe with associated host plants.

Population Number, Location and Species			Host Plant	n	Infection of Bacterial Symbiont (%)					Reference
					H	A	R	W	C	
1	Croatia/Turanj	MED (Q2)	*Cucumis sativus*	20	25	-	30	20	30	[39]
2	Croatia/Split	MED (Q1)	*Euphorbia pulcherrima*	20	35	-	20	10	-	This study
3	Croatia/Zadar ^¶^	MED (Q1 and Q2)	*Hibiscus* sp.	20	95	35	60	50	-	[41]
4	Montenegro/Bar	MED (Q2)	*Dipladenia sanderi*	20	65	85	100	40	-	
5	Montenegro/Lastva Grbaljska	MED (Q1)	*Cucumis sativus*	20	30	85	40	30	-	This study
6	Montenegro/Darza	MED (Q1)	*Cucumis melo*	20	95	-	10	10	-	
7	Macedonia/Doiran	MED (Q1)	*Lycopersicon esculentum*	20	100	-	-	40	20	
8	Macedonia/Gevgelija	MED (Q1)	*Cucumis melo*	20	100	-	5	60	-	
9	Israel	MED (Q2)	*Gossypium hirsutum*	20	-	100	75	20	-	[39]
10	Israel	MEAM1	*Gossypium hirsutum*	20	100	-	70	-	-	
11	Croatia/Split	*T*. *vaporariorum*	*Sonchus oleraceus*	20	5	75	-	-	-	
12	Croatia/Split^§^	*T*. *vaporariorum*	*Euphorbia pulcherrima*	10	50	80	-	-	-	[41]; This study
13	Montenegro/Podgorica	*T*. *vaporariorum*	*Sonchus oleraceus*	20	20	95	5	10	35	
14	Macedonia/Doiran	*T*. *vaporariorum*	*Lycopersicon esculentum*	20	25	100	-	-	-	This study
15	Macedonia/Grciste	*T*. *vaporariorum*	*Lycopersicon esculentum*	20	-	100	-	-	-	
16	Serbia/Trbusani	*T*. *vaporariorum*	*Cucurbita pepo*	20	90	100	-	-	-	
17	Serbia/Vranjska Banja	*T*. *vaporariorum*	*Gerbera* sp.	20	45	100	-	-	-	
18	Serbia/Opovo	*T*. *vaporariorum*	*Lycopersicon esculentum*	20	70	90	-	-	-	
19	Serbia/Zorka Subotica	*T*. *vaporariorum*	*Chrysanthemum* sp.	20	-	95	-	-	-	
20	Croatia/Brac-Supetar	*S. phillyreae*	*Punica granatum*	20	65	35	-	40	5	[41]
21	Croatia/Brac-Pucisca	*S. phillyreae*	*Punica granatum*	20	100	25	-	20	-	
22	Croatia/Opuzen	*S. phillyreae*	*Punica granatum*	20	85	95	-	55	5	
23	Croatia/Ljuta	*S. phillyreae*	*Punica granatum*	20	85	85	-	15	70	
24	Montenegro/Bar	*S. phillyreae*	*Punica granatum*	20	90	100	-	50	-	

n, total number of individual tested per population; H, *Hamiltonella*; A, *Arsenophonus*; R, *Rickettsia*; W, *Wolbachia*; C, *Cardinium*; -, zero. ^¶^ Population imported by trade from Italy (Rome, region Lazio). ^§^ Population imported by trade from Slovenia (Catez ob Savi). For additional information on imported whitefly populations, please refer to Skaljac et al. (2013) [41].

**Table 3:** Infection frequencies of different secondary bacterial symbiont combinations in the three whitefly species in this study.

Population Number, Location, and Species		Infection Frequencies of Bacterial Symbiont Combination (%)																								
		R	H	A	W	C	RH	RA	HA	HW	HC	AW	AC	WC	RHA	RHW	RAW	RWC	HAW	HAC	HWC	AWC	RHWC	RHAW	HAWC	No Infection
1	Croatia/Turanj MED (Q2)	10	10	-	5	15	15	-	-	-	-	-	-	5	-	-	-	5	-	-	-	-	5	-	-	35
2	Croatia/Split MED (Q1)	20	35	-	10	-	-	-	-	-	-	-	-	-	-	-	-	-	-	-	-	-	-	-	-	35
3	Croatia/Zadar^¶^ MED (Q1 and Q2)	-	20	-	-	-	15	-	15	5	-	-	-	-	-	25	5	-	-	-	-	-	-	15	-	-
4	Montenegro/Bar MED (Q2)	5	-	-	-	-	5	20	-	-	-	-	-	-	30	5	10	-	-	-	-	-	-	25	-	-
5	Montenegro/L. Grbaljska MED (Q1)	-	-	35	5	-	-	15	-	-	-	5	-	-	10	-	-	-	5	-	-	-	-	15	-	10
6	Montenegro/Darza MED (Q1)	-	75	-	-	-	10	-	-	10	-	-	-	-	-	-	-	-	-	-	-	-	-	-	-	5
7	Macedonia/Doiran MED (Q1)	-	55	-	-	-	-	-	-	25	5	-	-	-	-	-	-	-	-	-	15	-	-	-	-	-
8	Macedonia/Gevgelija MED (Q1)	-	40	-	-	-	-	-	-	55	-	-	-	-	-	5	-	-	-	-	-	-	-	-	-	-
9	Israel MED (Q2)	-	-	25	-	-	-	55	-	-	-	-	-	-	-	-	20	-	-	-	-	-	-	-	-	-
10	Israel MEAM1	-	30	-	-	-	70	-	-	-	-	-	-	-	-	-	-	-	-	-	-	-	-	-	-	-
11	Croatia/Split^§^ *T*. *vaporariorum*	-	-	70	-	-	-	-	5	-	-	-	-	-	-	-	-	-	-	-	-	-	-	-	-	25
12	Croatia§/Split *T*. *vaporariorum*	-	-	30	-	-	-	-	50	-	-	-	-	-	-	-	-	-	-	-	-	-	-	-	-	20
13	Montenegro/Podgorica *T*. *vaporariorum*	-	-	35	5	-	-	-	20	-	-	5	35	-	-	-	-	-	-	-	-	-	-	-	-	-
14	Macedonia/Doiran *T*. *vaporariorum*	-	-	75	-	-	-	-	25	-	-	-	-	-	-	-	-	-	-	-	-	-	-	-	-	-
15	Macedonia/Grciste *T*. *vaporariorum*	-	-	100	-	-	-	-	-	-	-	-	-	-	-	-	-	-	-	-	-	-	-	-	-	-
16	Serbia/Trbusani *T*. *vaporariorum*	-	-	10	-	-	-	-	90	-	-	-	-	-	-	-	-	-	-	-	-	-	-	-	-	-
17	Serbia/Vranjska Banja *T*. *vaporariorum*	-	-	55	-	-	-	-	45	-	-	-	-	-	-	-	-	-	-	-	-	-	-	-	-	-
18	Serbia/Opovo *T*. *vaporariorum*	-	5	25	-	-	-	-	65	-	-	-	-	-	-	-	-	-	-	-	-	-	-	-	-	5
19	Serbia/Z. Subotica *T*. *vaporariorum*	-	-	95	-	-	-	-	-	-	-	-	-	-	-	-	-	-	-	-	-	-	-	-	-	5
20	Croatia/Brac-Supetar *S. phillyreae*	-	25	-	10	-	-	-	20	10	-	5	-	5	-	-	-	-	10	-	-	-	-	-	-	15
21	Croatia/Brac-Pucisca *S. phillyreae*	-	60	-	-	-	-	-	20	15	-	-	-	-	-	-	-	-	5	-	-	-	-	-	-	-
22	Croatia/Opuzen *S. phillyreae*	-	5	5	-	-	-	-	35	-	-	10	-	-	-	-	-	-	40	-	-	-	-	-	5	-
23	Croatia/Ljuta *S. phillyreae*	-	10	5	-	-	-	-	15	-	-	-	5	-	-	-	-	-	-	50	-	5	-	-	10	-
24	Montenegro/Bar *S. phillyreae*	-	-	5	-	-	-	-	45	-	-	5	-	-	-	-	-	-	45	-	-	-	-	-	-	-

H, *Hamiltonella*; A, *Arsenophonus*; R, *Rickettsia*; W, *Wolbachia*; C, *Cardinium*; -, zero. ^¶^ Population imported by trade from Italy (Rome, region Lazio). ^§^ Population imported by trade from Slovenia (Catez ob Savi). For additional information on imported whitefly populations, please refer to Skaljac et al. (2013) [41].

## Supplementary Materials

The following are available online at www.mdpi.com/2075-4450/8/4/113/s1, Figure S1: Individual and multiple infections by secondary bacterial symbionts in five *B. tabaci* and six *T. vaporariorum* populations from Croatia, Montenegro, Macedonia, and Serbia. Each square represents one population and each column represents one type of symbiont; the 20 rows per table represent the 20 individuals tested per population. Gray fields indicate positive infection for the tested symbiont. Population number, country and geographical location, species, and number of tested individuals are indicated at the top of each table. Symbionts: R—*Rickettsia*, H—*Hamiltonella*, A—*Arsenophonus*, W—*Wolbachia*, C—*Cardinium*; Table S1. The whitefly populations used in this study.
