# Supplementary material for: Diversity and Phylogenetic Analyses of Bacterial Symbionts in Three Whitefly Species from Southeast Europe
Source: Insects. 2017 Oct 20;8(4):113. doi: 10.3390/insects8040113 (PMC5746796; doi:10.3390/insects8040113)
Supplement: Supplementary file 1 [file insects-08-00113-s001.pdf]

insects

MDPI

Supplementary Materials: Diversity and Phylogenetic Analyses of Bacterial Symbionts in Three Whitefly Species from Southeast Europe

Marisa Skaljac, Surapathrudu Kanakala, Katja Zanic, Jasna Puizina, Ivana Lepen Pleic and Murad Ghanim

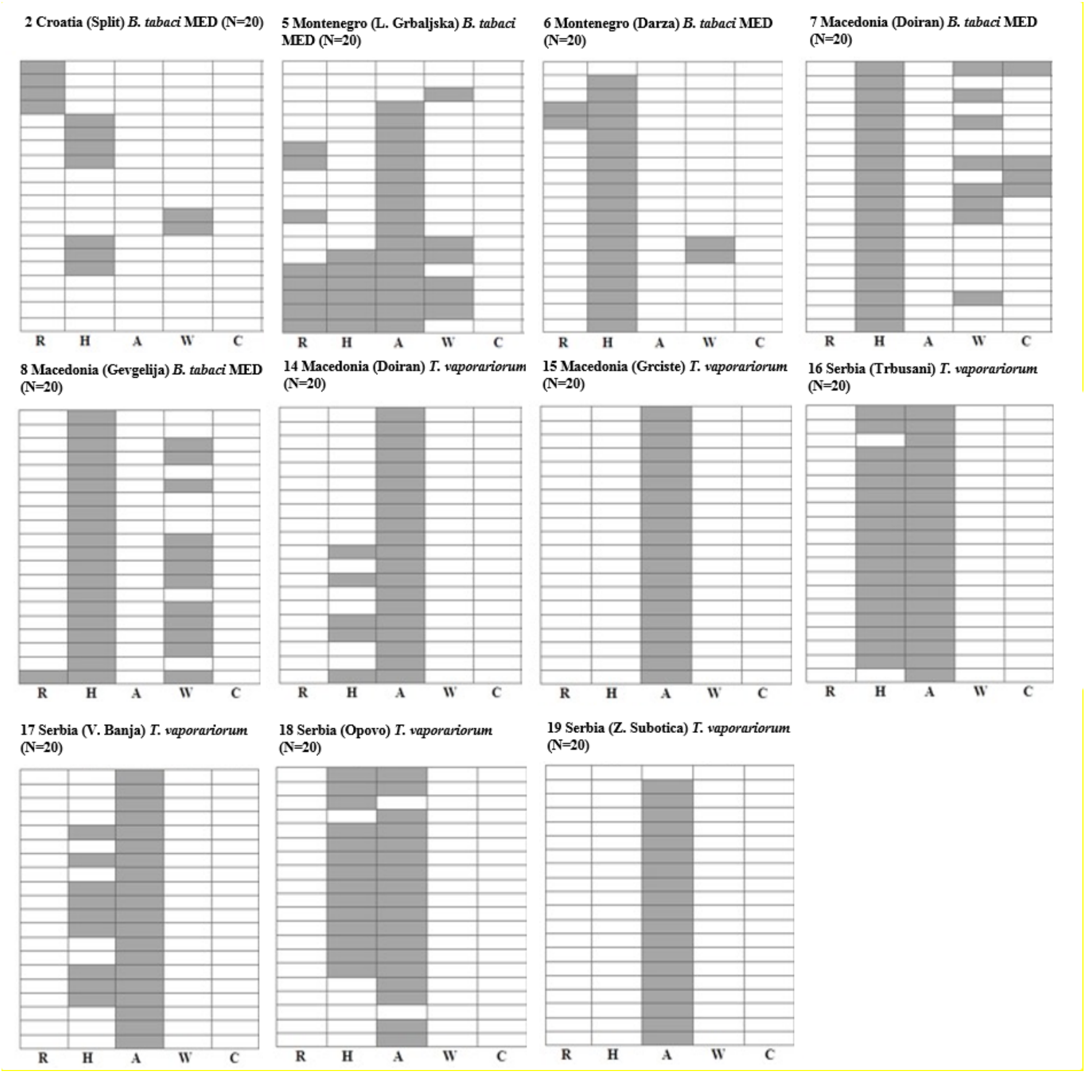

Figure S1. Individual and multiple infections by secondary bacterial symbionts in five *B. tabaci* and six *T. vaporariorum* populations from Croatia, Montenegro, Macedonia, and Serbia. Each square represents one population and each column represents one type of symbiont; the 20 rows per table represent the 20 individuals tested per population. Gray fields indicate positive infection for the tested symbiont. Population number, country and geographical location, species, and number of tested individuals are indicated at the top of each table. Symbionts: R—Rickettsia, H—*Hamiltonella*, A—*Arsenophonus*, W—*Wolbachia*, C—*Cardinium*.

Table S1. The whitefly populations used in this study.

Population Number	Country/Location	Coordinates	Species	Year
1	Croatia/Turanj	43°58'14" N, 15°24'59" E	MED (Q2)	2011
2	Croatia/Split	43°30'20" N, 16°30'24" E	MED (Q1)	2013
3	Croatia/Zadar[¶]	44°05'50" N, 15°15'46" E	MED (Q1 and Q2)	2011
4	Montenegro/Bar	42°05'08" N, 19°08'11" E	MED (Q2)	2011
5	Montenegro/Lastva Grbaljska	42°18'39" N, 18°48'16" E	MED (Q1)	2012
6	Montenegro/Darza	41°55'26" N, 19°14'08" E	MED (Q1)	2011
7	Macedonia/Doiran	41°14'30" N, 22°42'59" E	MED (Q1)	2012
8	Macedonia/Gevgelija	41°09'54" N, 22°30'19" E	MED (Q1)	2012
9	Israel	31°59'34" N, 34°49'06" E	MED (Q2)	2013
10	Israel	31°59'34" N, 34°49'06" E	MEAM1	2013
11	Croatia/Split	43°30'20" N, 16°30'24" E	*T. vaporariorum*	2013
12	Croatia/Split[§]	43°30'20" N, 16°30'24" E	*T. vaporariorum*	2010
13	Montenegro/Podgorica	42°27'43" N, 19°16'40" E	*T. vaporariorum*	2012
14	Macedonia/Doiran	41°14'30" N, 22°42'59" E	*T. vaporariorum*	2012
15	Macedonia/Grciste	41°15'25" N, 22°30'55" E	*T. vaporariorum*	2012
16	Serbia/Trbusani	43°55'28" N, 20°19'13" E	*T. vaporariorum*	2009
17	Serbia/Vranjska Banja	42°32'60" N, 22°00'00" E	*T. vaporariorum*	2010
18	Serbia/Opovo	45°04'05" N, 20°27'15" E	*T. vaporariorum*	2010
19	Serbia/Zorka Subotica	46°07'28" N, 19°38'35" E	*T. vaporariorum*	2010
20	Croatia/Brac-Supetar	43°22'50" N, 16°32'50" E	*S. phillyreae*	2011
21	Croatia/Brac-Pucisca	43°20'58" N, 16°44'03" E	*S. phillyreae*	2011
22	Croatia/Opuzen	43°00'45" N, 17°32'13" E	*S. phillyreae*	2011
23	Croatia/Ljuta	42°32'03" N, 18°22'43" E	*S. phillyreae*	2011
24	Montenegro/Bar	42°05'08" N, 19°08'11" E	*S. phillyreae*	2011

[¶] Population imported by trade from Italy (Rome, region Lazio). [§] Population imported by trade from Slovenia (Catez ob Savi). For additional information on imported whitefly populations, please refer to Skaljac et al. (2013) [41].

© 2017 by the authors. Submitted for possible open access publication under the terms and conditions of the Creative Commons Attribution (CC BY) license (http://creativecommons.org/licenses/by/4.0/).
